# Intra-Abdominal Abnormalities Associated with Polysplenia Syndrome

**DOI:** 10.5334/jbsr.1903

**Published:** 2019-10-02

**Authors:** Hadrien Renier

**Affiliations:** 1 Université de Liège, BE

**Keywords:** Polysplenia, Syndrome, Isomerism, Abdominopelvic Computed Tomography, Heterotaxy, Ambiguus

## Abstract

What to look for in case of polysplenia and/or unusual disposition of several intra-abdominal organs.

Anysmay, polysplenia syndrome is an unusual disposition of intra-abdominal organs and unlike situs inversus it’s a spectrum of abnormalities and not a single set.

## Report

An asymptomatic 62-year-old woman underwent abdominopelvic computed tomography (CT) for inaugural diabetes.

**Figure 1:**
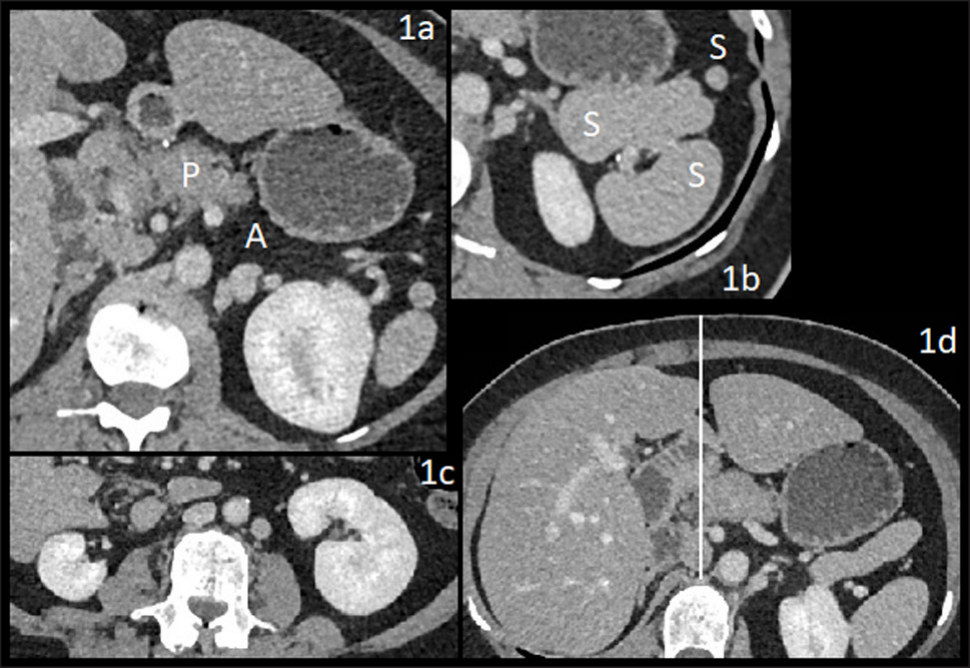


CT showed dorsal pancreatic (P) agenesis (A) (Figure 1a).

**Figure 2:**
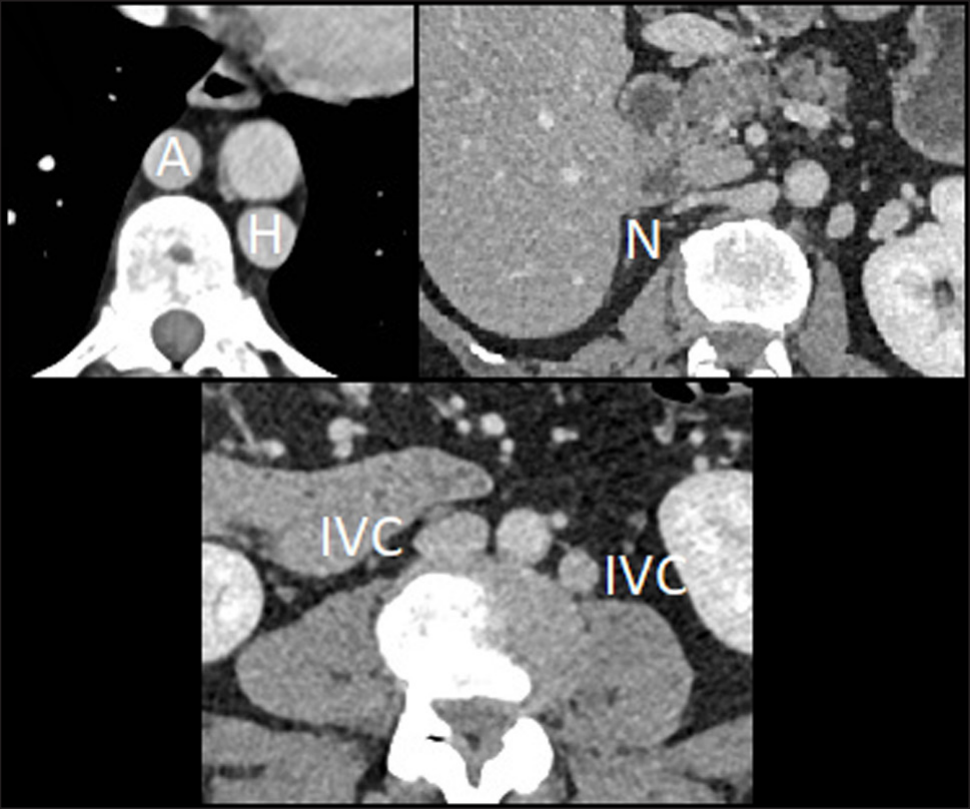


**Figure 3:**
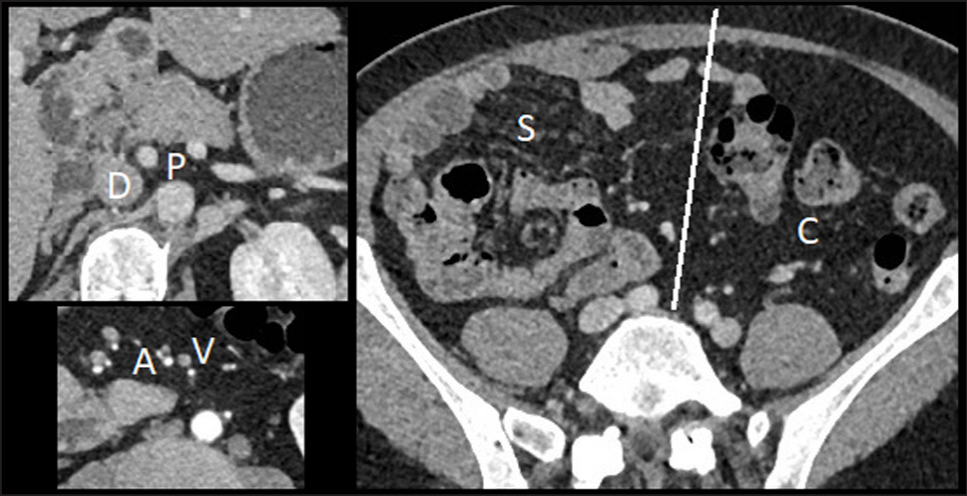


Incidental abnormalities were found, including:

– Four spleens (S), one adjacent to the stomach (Figure 1b).
– Right renal hypotrophy (Figure 1c).
– Midline falciform ligament (Figure 1d).
– Duplicated inferior vena cava system (IVC) with dilated azygos (A) and hemiazygos (H) continuation and no communication with hepatic veins (N) (Figure 2).
– Intestinal nonrotation: the small bowel was right-sided (S), the colon was left-sided (C), the superior mesenteric artery (A) was to the right of the vena (V), and there was no midline crossing by the duodenum (D) under the aorto-mesenteric junction (P) (Figure 3).

The diagnosis of type II diabetes was retained and after four months of metformin and insulin therapy, the rate of HbA1c was almost normalized. The remaining of the follow-up was unremarkable.

## Comment

Heterotaxy syndrome (HS) (or situs ambiguus) is the result of an early embryological developmental failure in which there is an abnormal arrangement of thoraco-abdominal organs. In contrast to situs inversus, HS is not characterized by a single set of abnormalities but rather a spectrum.

Polysplenia syndrome (PS) (or left isomerism) is the subtype of HS with features of bilateral left-sidedness. No single anomaly is pathognomonic but the association of a sufficient number allows the diagnosis. The commonest is the presence of multiple spleens, right- or left-sided, with a consistent relationship to the stomach.

As in the present case, the other intra-abdominal abnormalities include:

– midline liver with or without biliary abnormality,
– truncated pancreas with presence of the head and a variable portion of the body,
– azygos continuation of the IVC,
– midline or right-sided aorta,
– right-sided stomach and/or abnormalities of the mesentery rotation.

Compared to the other HS (i.e., right isomerism [or asplenia]) PS is often detected incidentally in adults. Indeed, it is associated with less severe or no congenital heart disease and no immune system deficiency [1].
